# Draft Genome Sequence of a Novel *Desulfobacteraceae* Member from a Sulfate-Reducing Bioreactor Metagenome

**DOI:** 10.1128/genomeA.01540-15

**Published:** 2016-01-14

**Authors:** Robert Almstrand, Ameet J. Pinto, Linda A. Figueroa, Jonathan O. Sharp

**Affiliations:** a Department of Civil and Environmental Engineering, Colorado School of Mines, Golden, Colorado, USA; b Infrastructure and Environment Research Division, School of Engineering, University of Glasgow, Glasgow, United Kingdom

## Abstract

Sulfate-reducing bacteria are important players in the global sulfur cycle and of considerable commercial interest. The draft genome sequence of a sulfate-reducing bacterium of the family *Desulfobacteraceae*, assembled from a sulfate-reducing bioreactor metagenome, indicates that heavy-metal– and acid-resistance traits of this organism may be of importance for its application in acid mine drainage mitigation.

## GENOME ANNOUNCEMENT

Sulfate-reducing bacteria (SRB) are widespread and abundant in nature and of considerable commercial importance, ranging from their role in corrosion (1) to their application in sulfate-reducing bioreactors (SRBRs) for treatment of mining-influenced waters (2). With few exceptions (3, 4), most characterized SRB are not tolerant to acidic conditions typical of acid mine drainage and require circumneutral pH (5) in order to efficiently reduce sulfate and promote metal immobilization in SRBRs. Here, we present a draft genome of a sulfate-reducing bacterium of the family *Desulfobacteraceae*, which was assembled from a sulfate-reducing bioreactor metagenome. Based on phylogenetic analyses using 16 ribosomal proteins (6), the genome of this organism clustered with *Desulfatirhabdium butyrativorans*, a butyrate-oxidizing SRB isolated from an anaerobic sludge blanket reactor treating industrial wastewater (7).

SRBR DNA was extracted using the Power Soil DNA isolation kit (MoBio Laboratories, Carlsbad, CA, USA). The genomic DNA library was prepared using an Illumina TruSEQ DNA library kit and sequenced on an Illumina HiSEQ 2500 paired-end flow cell (2 × 125-bp read length) using V4 Chemistry at the Genomics and Microarray Core, University of Colorado, Denver. IDBA-UD version 1.1.1 (8) was used to assemble the reads along with four additional samples. The resulting scaffolds were binned using CONCOCT (9), followed by extraction of mapped reads and reassembly using IDBA-UD. Following reassembly, contigs less than 1 kb and any contigs showing breaks in coverage profile were removed. The resulting genome bin contained 193 contigs with a genome size of 6.09Mbp (*N*_50_ = 33,167 bp) and a GC content of ~50%. CheckM (10) indicated a genome completeness of 97.8% with likely contamination of 0.64% and no strain heterogeneity. Comparison of the draft genome to complete reference genomes of *Desulfatibacillum alkenivorans* AK-01, *Desulfobacula toluolica* Tol2, *Desulfobacterium autotrophicum* HRM2, and *Desulfococcus oleovorans* Hxd3 using the Genome-to-Genome Distance Calculator (http://ggdc.dsmz.de) indicated an average nucleotide identity of 70.7 ± 0.4% to the reference genomes. Gene calling was performed using Prodigal (11), and the genes were annotated against the KEGG database (12) using RAPSearch2 (13). The genome consisted of 3,694 coding regions with 3,463 matches to the KEGG database.

The assembled *Desulfobacteraceae* genome appears capable of nitrogen fixation and urea-utilization, containing a urea transport system not encountered in the reference genomes. Similar to the reference genomes, this draft contains a two-component system (HydH-HydG), previously implicated in bacterial metal tolerance (14, 15). A unique feature of this draft genome when compared to other *Desulfobacteraceae* members is a γ-aminobutyrate (GABA) shunt involved in microbial acid resistance (16), raising the possibility that this genome represents a more acid-tolerant member of the family *Desulfobacteraceae* with potential interest for the mitigation of metal-laden, acidic waters.

### Nucleotide sequence accession numbers.

This whole-genome shotgun project has been deposited in GenBank under the accession number LKPX00000000. The version described in this paper is the first version, LKPX01000000.
